# The Impact of Focused Hip Ultrasound Training on Imaging Quality in Infants With Hip Dysplasia

**DOI:** 10.7759/cureus.74787

**Published:** 2024-11-29

**Authors:** Ahmad Alawadhi, Kenan S Basha, Jalal Al-Shryda, Tahani Al Ali, Ajay P Dsouza, Hayder S Abdulhadi, Amar H Khamis, Ibrar Majid, Sattar Alshryda

**Affiliations:** 1 College of Medicine, Mohammed Bin Rashid University of Medicine and Health Sciences, Dubai, ARE; 2 Department of Surgery, Collm Klinik Oschatz, Oschatz, DEU; 3 Department of Orthopedics, Al Jalila Children's Specialty Hospital, Dubai, ARE; 4 Department of Radiology, Al Jalila Children's Specialty Hospital, Dubai, ARE; 5 Department of Orthopedics, Rashid Hospital, Dubai, ARE; 6 Department of Pediatric Orthopedics, Al Jalila Children's Specialty Hospital, Dubai, ARE; 7 Department of Pediatric Orthopedics and Trauma, Al Jalila Children's Specialty Hospital, Dubai, ARE

**Keywords:** developmental dysplasia of the hip ( ddh ), graf method, hips, neonatal screening, pavlik harness, pediatric orthopedics, pediatric ultrasound, ultrasound

## Abstract

Background: The orthopedic department at Al Jalila Children's Specialty Hospital (AJCH) was opened in April 2018. A focused hip ultrasound training course was conducted in April 2019 to improve hip ultrasound imaging quality.

Objectives: This study aims to evaluate the impact of focused training courses on predefined image quality criteria of infant hip ultrasound in the context of developmental hip dysplasia. It also seeks to measure the inter- and intra-rater agreement among various disciplines.

Methods: A retrospective review of 120 hip ultrasound images (60 infants) was performed between April 2018 and April 2020. Based on internationally agreed criteria, 60 hip images obtained before the course were compared to another 60 hip images obtained after the course. Inter-rater and intra-rater agreements were also evaluated using intraclass correlation (ICC).

Results: The study evaluated the impact of a focused training course on the quality of infant hip ultrasound images for developmental dysplasia of the hip. Image quality significantly improved after the training, with optimal images increasing from 48% to 82% (P<0.001). Logistic regression confirmed the training's positive effect, highlighting its clinical and statistical significance. The study has also demonstrated excellent agreement among raters for alpha and beta angles, as reflected by ICC statistics. The agreement for alpha angles was notably higher than for beta angles (ICC 0.970 vs. 0.904; P<0.0001). However, inter-rater agreement for hip types, assessed using kappa statistics, was moderate (κ = 0.512) and targeted to address a limited shortfall or gaps in services.

Conclusion: The study confirms the value of focused training in improving the quality of care. This training should be carefully planned and targeted to address limited shortfalls or gaps in services in other areas of service delivery.

Level of evidence: The study is a retrospective cohort with evidence level II.

## Introduction

Developmental dysplasia of the hip (DDH) describes a spectrum of hip abnormalities ranging in severity from mild hip dysplasia that is detectable on radiological imaging only to a complete and irreducible dislocated hip that is obvious clinically [1]. It is considered one of the most common pediatric hip conditions, with 8% of newborns having hip dysplasia and one in 1000 having a dislocated hip at birth worldwide [2]. A similar incidence was reported in the United Arab Emirates. Moosa and colleagues reported that 3.17 per 1000 newly born children had radiologically confirmed hip dysplasia, with 2.7 per 1000 of them clinically detectable. Several risk factors have been linked to DDH; however, breech presentation, positive family history, and female gender are the widely accepted independent risk factors [3-6].

Early diagnosis of DDH is essential for a simpler treatment and a better long-term outcome. If the diagnosis is made in the first six weeks of life, the treatment is simple, and the success rate is very high [2,7]. This involves a device called the "Pavlik harness" that is worn by the child for nearly six weeks when the hips become normal. The success rate is over 90% [8].

Late diagnosis prevents the application of simple treatment methods due to the progressive tightening of soft tissues, narrowing of the joint capsule, and the inability to achieve reduction. Additionally, the bony structures around the hip socket remain flattened, failing to maintain joint stability. As a result, surgical interventions become necessary to achieve and maintain joint reduction. These procedures may include open reduction of the hip joint, muscle lengthening, and pelvic and femoral osteotomies, followed by immobilization in a hip spica cast for up to three months [9,10].

Hip ultrasound using Graf methods has been proven to be the best method in the early detection of DDH [2,11-15]. The hips are graded based on validated anatomical landmarks and angles on a single image in the "standard plane" [16]. The types of hips according to the Graf method will be discussed in the succeeding section. Some countries, such as Germany, Austria, and Switzerland, adopted a universal hip screening program for all newly born children for early detection and effective treatment. Other countries adopted a screening program for high-risk babies (selective screening program). It is still being debated which approach is better [2,14].

Although hip ultrasound has been identified as the gold standard, it is relatively specialized, and not many radiologists have been specifically trained for it. Many radiologists have self-learned rather than specifically trained, creating a widespread suboptimal practice [17,18]. Two studies from the Middle East highlighted this fact. The first study from Tehran University of Medical Science stated that "screening protocols need to be improved with the help of trained paediatricians and other health professions [17]. The second study from King Khalid University Hospital, Riyadh, indicated that many DDH cases are diagnosed late due to poor national screening protocols [18].

Al Jalila Children's Specialty Hospital (AJCH) was established to provide high-standard healthcare for children in the region. However, it was immediately apparent that the hip screening for the DDH did not meet the aspiration of providing top-level healthcare. Therefore, action plans to improve the situation were put in place. One initiative was to run a Graf hip ultrasound course locally. The first course was held in April 2019 at AJCH, and 23 clinicians were trained.

The purpose of the study is to evaluate the impact of the focused hip ultrasound training using Graf methods on the quality of diagnostic imaging in children with DDH and on the inter- and intra-agreement among staff before and after the course.

## Materials and methods

This is a retrospective study of two groups of patients with DDH who underwent hip ultrasound; one group consists of 30 patients (60 hips) who were scanned before a focused training course on hip sonography and another matching group of 30 patients (60 hips) after the course. The study findings have been written in accordance with the Strengthening the Reporting of Observational Studies in Epidemiology (STROBE) guidelines [19].

Eligibility criteria for participants are consequent infants that were scanned for DDH between April 1, 2018, and April 1, 2020, with an age of no more than six months. The first scan was included in the analysis as it is the decision-making scan to treat, follow up, or discharge a patient. Exclusion criteria included patients who had ultrasound outside AJCH, ultrasound for conditions other than DDH, or incomplete scans. Data were collected retrospectively through electronic medical records (Cerner). The patient list was generated through the IT department.

The study exposure was a two-day focused training course on diagnosing and treating children with DDH using the Graf methods in April 2019. It was undertaken by 23 candidates, of whom there were two orthopedic surgeons, two radiologists, and three sonographers who worked at AJCH. All images included in the study were performed by radiologists or sonographers. The images were anonymized and circulated to the raters who were not previously involved in producing or reporting them. The primary outcome of the study is to compare imaging quality before and after the training course using a well-defined criterion consisting of 10 variables mentioned in Table 1 below [14-16,20].

**Table 1:** Graf infant hip classification auditing tool. This tool has served as quality assurance criteria for high-standard hip ultrasound imaging. The table was reproduced from the Graf hip course unpublished material.

Part A
1. Is the image in the standard plane?
2. Is the lower limb of the ileum visualized?
3. Is the labrum visualized?
4. Has the alpha angle correctly measured?
5. Has the beta angle correctly measured?
6. Has the hip type been identified correctly?
7. Report consistent with images
Part B
1. Has an appropriate transducer been used?
2. Has the appropriate image size been displayed?
3. Have the appropriate gain settings been used?

Each criterion was assigned a score of 1. The total score was then recorded as a binary outcome: "Meet the standard" or "Do not meet the standard." Any scan that did not achieve a full score was considered "Does not meet the standard." The difference between the total scores of the two groups was tested for significance using a logistic regression model, and the binary outcome status was tested using the chi-square.

**Figure 1:**
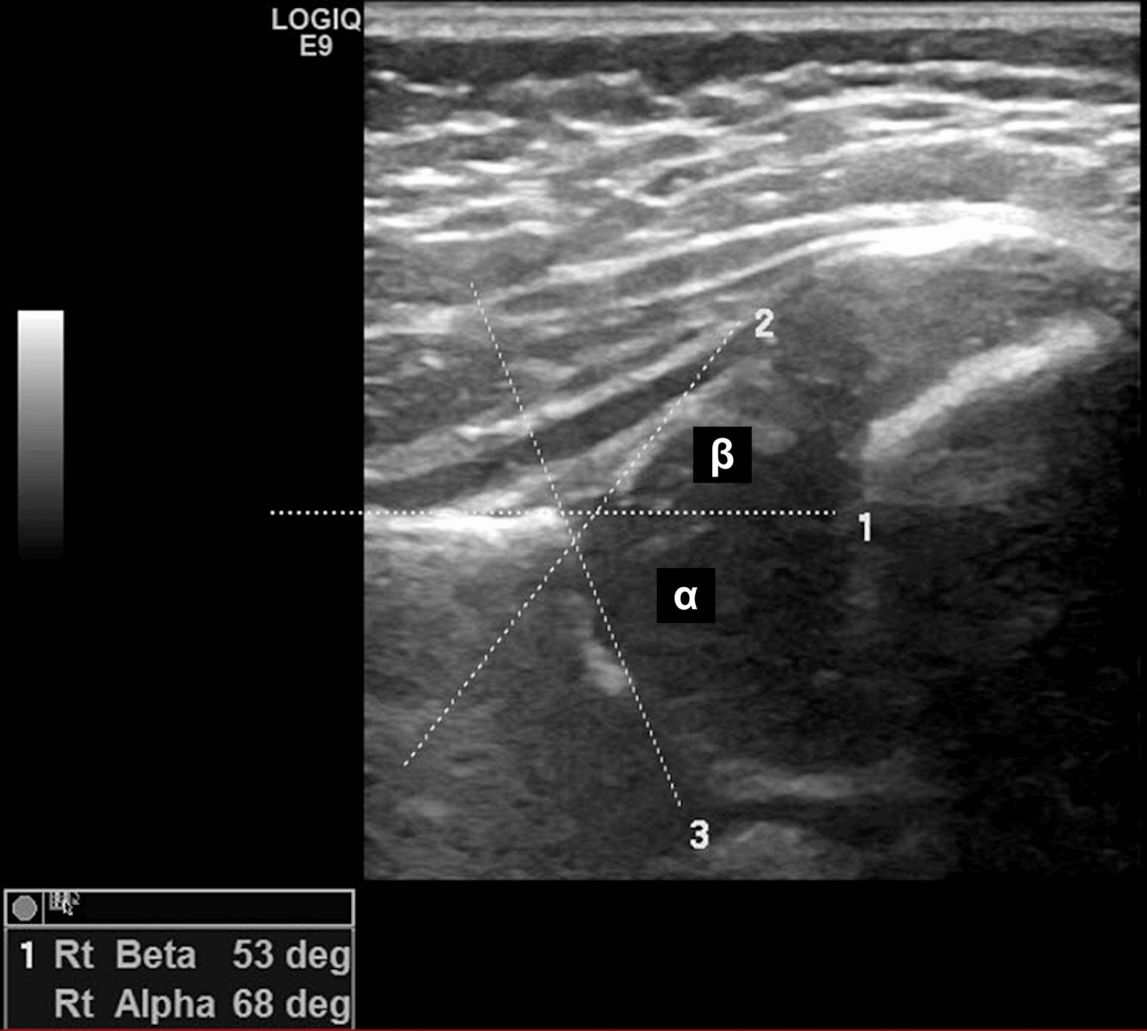
Hip ultrasound image. The image shows the correct measurement of alpha and beta angles. Ensuring the accuracy of an image involves two crucial steps. The first step is identifying eight anatomical landmarks on the image: the chondro-osseous border, femoral head, synovial fold, joint capsule, labrum, the cartilage portion of the roof, the bony portion of the roof, the bony rim (also known as the turning point between the bony roof's concavity and convexity), and the ilium. The second step is completing the usability checklist, which ensures the identification of three key elements: the lower limb of the acetabular roof (typically the brightest and most prominent lower end of the bony roof), the midportion of the ilium, and the labrum. If any of these elements are missing or not clearly visible, the sonogram is considered invalid and should not be used.

The training was repeated in October 2020 on a one-on-one basis with a medical student. While the student did not perform any scanning, he participated in reading the images for inter-rater and intra-rater comparisons. Three raters (a consultant radiologist, a senior sonographer, and a medical student) who were trained in the Graf method were compared in their assessments of three key outcomes on a hip ultrasound image: the alpha angle (α), beta angle (β), and hip type (Figure 1).

To minimize confirmation bias, the raters were blinded to patient details when measuring the inter-observer reliability. Each examiner was asked independently to identify the above three variables (alpha and beta angles and types of hips) of images without reading the report or identifications. For intra-observer reliability, the raters were asked to measure the same variables on the same images that were re-ordered a week apart. These raters were also compared with the reported figures in the original ultrasound reports.

The inter- and intra-raters' agreement of α and β angles, being quantitative variables, was measured using intraclass correlation coefficient (ICC) analysis. Kappa (κ) analysis was used for the types of hips as they are ordinal variables [21,22].

The sample size was reached by selecting all consecutive patients a year before and a year after the focused hip training. The number was adjusted to ensure matching groups after excluding patients who did not meet the inclusion criteria.

Data were collected using an Excel sheet (Microsoft Corporation, Redmond, Washington), which was then imported into IBM SPSS Statistics for Windows, Version 25 (Released 2017; IBM Corp., Armonk, New York) [23]. Angles were measured in degrees. Standard criteria were recorded as a binary variable: yes, if an image met the quality standard criterion, and no, if not. The types of hips were recorded as ordinal variables, according to Graf grades: (Type I, Type IIa, Type IIb, Type IIc, Type D, Type III, and Type IV) (Table 2).

**Table 2:** Graf infant hip classification. This is the most widely used infant hip classification to grade developmental dysplasia of the hip [1].

Type	Alpha Angle (α)	Beta Angle (β)	Notes
Ia	> 60°	< 55°	Normal hip (at any age). This grade is further divided into (Ia; β<55°) and (Ib; β> 55°). The significance of this subdivision is not yet established. The patient does not need follow-up.
Ia	> 60°	> 55°	
IIa	50-59°	< 77°	If the child is < 3 months. This may be physiological and does not need treatment; however, follow-up is required.
IIb	50-59°	< 77°	> 3 months, delayed ossification.
IIc	43-49°	< 77°	This is further categorized into stable and unstable based on the provocation test. If the β angle increases above 77° during the provocation test, the hip is classified as type IIc unstable. If the β angle does not exceed this threshold, it is classified as type IIc stable.
D	43-49°	> 77°	This is the first stage in which the hip becomes decentred (subluxated). It used to be called IId, but for the above reason, it is now a separate stage.
IIIa	< 43°	Irrelevant	The dislocated femoral head with the cartilaginous acetabular roof is pushed upwards. This is further divided into IIIa and IIIb depending on the echogenicity of the hyaline cartilage of the acetabular roof (usually compared to the femoral head), which reflects the degenerative changes. Although the β angle is not important in the diagnosis, it may be useful to monitor the progress of the treatment in the early stages.
IIIb	< 43°	Irrelevant	
IV	< 43°	Irrelevant	The dislocated femoral head with the cartilaginous acetabular roof is pushed downwards.

Data were tested for normality using the Shapiro-Wilk/Kolmogorov-Smirnov test as appropriate. A cross-tabulation will be used to examine the independence between related categorical variables, and statistical tests were performed using the chi-square or exact Fisher test as appropriate. A P-value of less than 0.05 was considered significant.

Agreement and intra- and inter-rater reliability were tested using ICC for angle measurements alpha and beta (α, β) and kappa (κ) analysis for hip type classification. The applied rules were summarized in Tables 3, 4 [21,24].

**Table 3:** Rules of intraclass correlation coefficient (ICC) analysis. Source: [20]

Agreement/Reliability	ICC
Excellent	x ≥ 0.9
Good	0.9 > x ≥ 0.75
Moderate	0.75 > x ≥ 0.5
Poor	x < 0.5

**Table 4:** Rules of kappa analysis. Source: [24]

Agreement/Reliability	Kappa (κ)
Perfect to almost perfect	1 > x ≥ 0.80
Substantial	0.80 > x ≥ 0.60
Moderate	0.60 > x ≥ 0.40
Fair	0.40 > x ≥ 0.20
None to slight	0.20 > x ≥ 0.01
No agreement	x < 0

## Results

A total of 307 participants were identified from the AJCH electronic medical record, and 95 were included after applying exclusion criteria. In total, 60 matching participants were selected, with 30 having their hip ultrasounds before the training course and 30 after. The participants were primarily female (60%), with a mean age of 1.9 months (SD 1.32), an average weight of 7.8 kg (SD 2.09 kg), and 62% identified as Emirati nationals.

Difference in image quality after training

**Figure 2:**
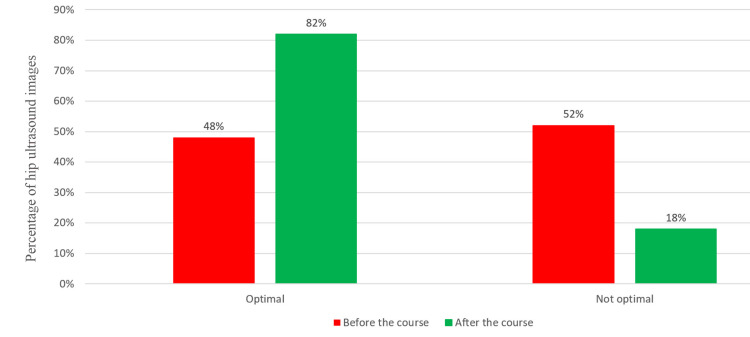
The improvement in the quality of infant hip ultrasound images after the focused training course. After the course, 82% of the images were considered optimal, compared to 48% before the course. Images are created by the authors.

The quality of images improved significantly after the focused training. Before the course, 48% of the images were considered optimal, compared to 82% after the course (Figure 2). This improvement was both clinically and statistically significant (FET: P<0.001). This finding was further confirmed by logistic regression analysis, with the focused training course as the dependent variable.

Inter-rater and intra-rater agreement

Agreement among raters for alpha and beta angles was excellent, as indicated by ICC statistics. The raters' agreement on alpha angles was higher than on beta angles (ICC 0.970 vs. 0.904; P<0.0001) (Table 5). The agreement among raters for hip types, analyzed using inter-rater kappa agreement, was moderate (κ = 0.512) (Table 6).

**Table 5:** Inter-rater agreement of alpha and beta angles. Agreement among raters for alpha and beta angles was excellent using ICC statistics. ICC: Intraclass Correlation Coefficient

Variable	Intraclass Correlation	95% Confidence Interval		F Test With True Value 0	
		Lower Bound	Upper Bound	Value	Significance
Αlpha angle	0.970	0.953	0.982	33.827	<0.001
Βeta angle	0.904	0.847	0.942	10.397	<0.001

**Table 6:** Inter-examiner error in hip classification. Agreement among raters for hip types was analyzed using inter-rater kappa agreement. The overall agreement was moderate.

Examiners	Kappa Value	Asymptotic Standard Error	Approximate Significance
Consultant Radiologist and Senior Sonographer	.591	.084	< .001
Consultant Radiologist and Medical Student	.399	.089	< .001
Senior Sonographer and Medical Student	.547	.082	< .001
Overall	.512	-	-

Intra-rater agreement was consistently excellent across all variables, with ICC and κ values both greater than 0.9, as shown in Tables 7, 8.

**Table 7:** Intra-rater agreement for alpha and beta angles. The intra-rater agreement was excellent for alpha and beta angles.

Examiner	Variable	Intraclass Correlation	95% Confidence Interval		F Test With True Value 0	
			Lower Bound	Upper Bound	Value	Significance
Consultant Radiologist	Alpha	.985	.974	.991	65.665	< .001
	Beta	.922	.867	.954	12.866	< .001
Senior Sonographer	Alpha	.948	.911	.969	19.183	< .001
	Beta	.911	.848	.948	11.191	< .001
Medical Student	Alpha	.999	.998	.999	843.617	< .001
	Beta	.995	.992	.997	215.600	< .001

**Table 8:** Intra-rater agreement for the types of hips. The intra-rater agreement on hip types was excellent among the senior radiologist and medical student, while it was moderate for the sonographer.

Examiners	Kappa Value	Asymptotic Standard Error	Approximate Significance
Consultant Radiologist	.905	.052	< .001
Senior Sonographer	.516	.091	< .001
Medical Student	1.000	.000	< .001

## Discussion

**Figure 3:**
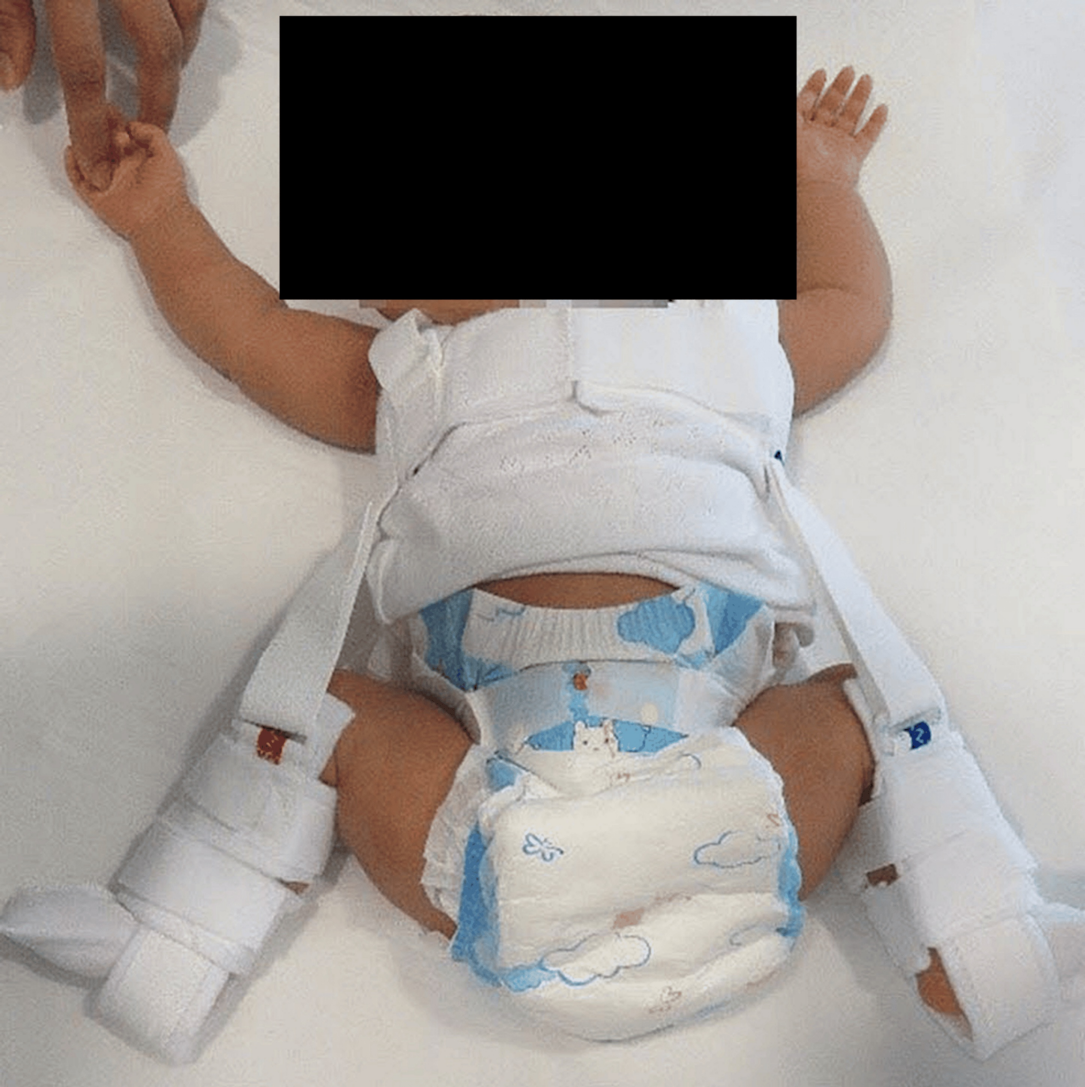
A child wearing a Pavlik harness. Early diagnosis of developmental dysplasia of the hip can lead to simple treatment, such as a Pavlik harness or similar devices, and avoiding major surgery.

**Figure 4:**
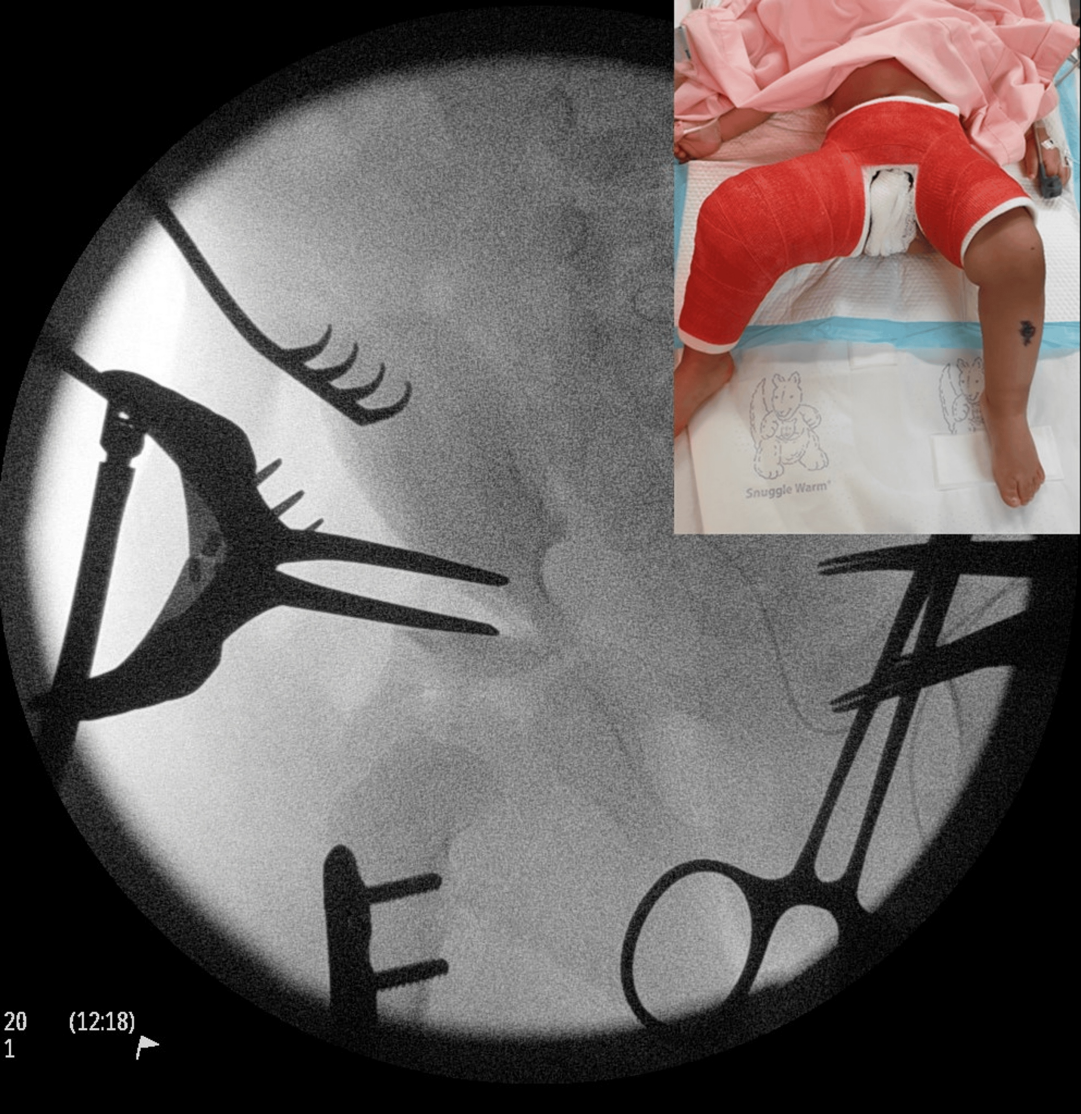
A compound image of open reduction of a dislocated hip in a child who presented late. Late presentation of hip dysplasia often involves major surgery of open reduction of the hip, pelvic, and femoral osteotomy. After surgery, the child is put in a hip spica (inset) for eight weeks. These could be prevented by early, high-quality hip ultrasound.

DDH is one of the most common pediatric orthopedic hip problems that attract substantive debates with regard to etiology, diagnosis, and treatment. Early diagnosis of DDH within the first six weeks of life can be treated with a device called the "Pavlik harness" that is worn by the child for nearly six weeks when most hips become normal (Figure 3). The success rate is over 90% [8]. Whereas a late diagnosis leads to major surgery of open reduction of the hip, pelvic, femoral osteotomies, and hip spica for as long as three months (Figure 4).

Graf's work in the 70s and 80s has been instrumental in advancing our understanding about the pathoanatomy of the condition. His work led to the development of the Graf hip classification and the emergence of hip ultrasound as the gold standard for early diagnosis [11-13,17,25,26].

Unfortunately, it takes a very long time for advanced bench research to translate to clinical practice, and we still witness that many centers have not caught up with the latest advances. This is what one of our findings has confirmed. Less than 50% of the images met the quality standard criteria that Professor Graf had set.

The concept of hip sonography, according to Graf, is very simple and can be taught over a two- or three-day course to a wide spectrum of clinicians. Over 100 similar focused courses have been delivered by Professor Graf in 20 countries; however, to the best of our knowledge, the impact of these courses on the quality of care has not been evaluated to the extent that we have done in this study. A relatively similar Japanese study was published in 2017 in which authors evaluated the inter- and intra-rater agreement among candidates who attended the Graf hip course, but they did not assess the impact of the course on the quality of images [27].

The improvement in the quality of images after the focused training course has been pleasantly impressive but still not perfect. An improvement in the percentage of images that meet the standard from 48% to 82% is an obvious success. This is despite the fact that we have been very strict in applying the standard criteria when we even rejected images that failed a single criterion and they would have been readily accepted as optimal in other centers.

Another interesting finding that our study has confirmed is that Graf methods have good inter- and intra-rater agreements regardless of the experience of the raters. Our findings showed excellent agreement in measuring alpha and beta angles and good agreement in identifying the types of hips among a senior radiologist, a sonographer, and a medical student who were trained in the method. What is even more interesting is that the quality of images improved after the course, which in turn translated to an even higher agreement of raters on post-course images.

Shirai et al., who reported the aforementioned Japanese study, observed a similar high inter-reader reliability in terms of the ultrasound images acquired between inexperienced examiners (good; ICC value of 0.75 for the α angle and excellent; ICC value of 0.86 for the β angle) [26].

Our findings are supported by other studies, although not to the same magnitudes of agreement [28,29].

DDH is one of the very few conditions on which the world approach is polarized. English-speaking countries such as the UK, USA, Canada, and Australia have adopted a selective screening in which high-risk babies are only offered hip ultrasound. This is in contrast to German-speaking countries such as Germany, Austria, and Switzerland, which adopted a universal screening program in which every newborn child is offered hip ultrasound screening. It is beyond the scope of this work to show which approach is superior, but our work supports the notion that hip ultrasound is simple to understand and learn. Even inexperienced professionals can be trained to safe competency in fewer than three days and should not be a barrier against universal screening.

Our study has several strengths. First, it followed strict scientific ways that were enforced by the joint agreement between a recognized educational institute and a university hospital. Inputs from academic staff such as statisticians and epidemiologists intertwined with advanced clinical skills in a teaching hospital. One of the senior researchers has been a hip ultrasound trainer using Graf methods for over 10 years. Second, being a newly opened hospital, it was easier to implement high-standard practices, which could explain the adherence to the trained standards. Third, guided by the university statistician, the use of appropriate statistical tests that are often lacking in similar other studies. Fourth, there is a close proximity between the date of the course and the study. Last but not least, the involvement of Professor Graf in person in the training course would inspire staff to adhere to his teachings. Although proportionate, the number of candidates is relatively low, and the study would have benefited from a bigger number of participants.

The study identified areas of future research projects. This study is the first phase in evaluating DDH service in the UAE. The second phase will expand on the impact of the course on treatment outcomes, which needs a long-term follow-up of a minimal five-year follow-up. Another is the utilization of artificial intelligence in measuring alpha and beta angles, along with identifying hip types and recommending treatment in a similar fashion to the ECG machine. It has also laid the groundwork for the Global DDH study, which is being planned by the ICODE experts (http://www.icode.expert/INCLUDE/default_new.asp).

Our study has a few limitations. The study is retrospective, with a relatively small number. It focuses primarily on the immediate impact of the training course on the quality of hip ultrasound images. However, it does not assess the long-term outcomes of the training on clinical practice and patient outcomes. A longer follow-up period, ideally five years or more, would be necessary to evaluate whether the improvements in image quality translate into better clinical outcomes. The involvement of Professor Graf in the training could have influenced the results, as participants might have been particularly motivated to adhere to his teachings. This might not reflect the typical outcomes of similar training programs without his direct involvement.

Healthcare is an ever-evolving field, necessitating changes over time. We cannot treat patients the same way we did a decade ago. Continuous learning and professional development are essential to keeping knowledge and skills current. Institutes and clinicians must stay attuned to advancements and actively seek to learn and embrace them. This study examined how a two-day course could enhance care for thousands of children. Hospitals should support staff in attending or organizing such courses, as the impact can be significant.

DDH exemplifies a condition in which substantial advancements and paradigm shifts have occurred in assessment and early treatment. While many countries and health centers have moved toward universal screening, most still rely on clinical surveillance and selective screening. As demonstrated in this study, focused training courses for healthcare providers can effectively build capacity toward universal screening, consistently improving the quality of treatment for our children.

## Conclusions

The study evaluated the impact of a focused training course on the quality of infant hip ultrasound images for DDH. Image quality significantly improved after the training, with optimal images increasing from 48% to 82% (P<0.001). Logistic regression confirmed the training's positive effect, highlighting its clinical and statistical significance. Excellent agreement among raters for alpha and beta angles was demonstrated; however, agreement among raters for hip types was moderate (κ = 0.512).
